# Phosphoproteomic analysis identifies differentially expressed phosphorylation sites that affect muscle fiber type in pigs

**DOI:** 10.3389/fnut.2022.1006739

**Published:** 2022-12-22

**Authors:** Yu He, Xiaofan Tan, Hongqiang Li, Zhiwei Yan, Jing Chen, Ruixue Zhao, David M. Irwin, Wangjun Wu, Shuyi Zhang, Bojiang Li

**Affiliations:** ^1^Department of Animal Genetics, Breeding and Reproduction, College of Animal Science and Veterinary Medicine, Shenyang Agricultural University, Shenyang, China; ^2^Hebei Key Laboratory of Specialty Animal Germplasm Resources Exploration and Innovation, College of Animal Science and Technology, Hebei Normal University of Science and Technology, Qinhuangdao, China; ^3^Department of Laboratory Medicine and Pathobiology, University of Toronto, Toronto, ON, Canada; ^4^Department of Animal Genetics, Breeding and Reproduction, College of Animal Science and Technology, Nanjing Agricultural University, Nanjing, China

**Keywords:** pigs, phosphoproteomic, differentially expressed phosphorylation sites, muscle fiber types, meat quality

## Abstract

Skeletal muscle of livestock is composed of both fast- and slow-twitch muscle fibers, which are key factors in their meat quality. However, the role of protein phosphorylation in muscle fiber type is not completely understood. Here, a fast-twitch (biceps femoris, BF) and slow-twitch (soleus, SOL) muscle tissue sample was collected from three male offspring of Duroc and Meishan pigs. We demonstrate that the meat quality of SOL muscle is significantly better than that of BF muscle. We further used phosphoproteomic profiling of BF and SOL muscles to identify differences between these muscle types. A total of 2,327 phosphorylation sites from 770 phosphoproteins were identified. Among these sites, 287 differentially expressed phosphorylation sites (DEPSs) were identified between BF and SOL. GO and KEGG enrichment analysis of proteins containing DEPSs showed that these phosphorylated proteins were enriched in the glycolytic process GO term and the AMPK signaling pathway. A protein-protein interaction (PPI) analysis reveals that these phosphorylated proteins interact with each other to regulate the transformation of muscle fiber type. These analyses reveal that protein phosphorylation modifications are involved in porcine skeletal muscle fiber type transformation. This study provides new insights into the molecular mechanisms by which protein phosphorylation regulates muscle fiber type transformation and meat quality in pigs.

## Introduction

Skeletal muscle, which is a heterogeneous tissue, accounts for approximately 40% of body mass in mammals (1). Muscle fibers are the main component of skeletal muscle and are classified as type I, IIA, IIX, and IIB based on the predominant myosin heavy chain isoform (2, 3). Slow muscle fiber is mainly composed of myosin heavy chain I (MyHC I), while fast muscle fiber contains three types of myosin heavy chains: IIA (MyHC IIA), IIX (MyHC IIX), and IIB (MyHC IIB) (2, 4). Muscles with different types of fibers also display differences in metabolic properties, which have different mitochondrial content (5). Previous studies have shown that muscle fiber type is closely related to meat quality (6–8). For example, Kang et al. demonstrated that high level of MyHC I is associated with improved pork meat quality through changes in pH, tenderness, and drip loss (7). In recent years, as the living standards of consumers have improved, they have demanded better quality pork. Therefore, control of muscle fiber type has become an approach to improve the quality of pork. Previous reports have found that muscle fiber type is influenced by many regulatory factors, including hormones (9), nutrients (10), genes (11), non-coding RNAs (12, 13), and calcineurin (14). Specifically, a recent study reported that dihydromyricetin regulates the conversion of fast to slow muscle through AMPK signaling, thereby improving pork quality (10). Shen et al. found that miR-152 improves pork quality by regulating glycolytic activity (12). Nevertheless, mechanisms that govern the type of muscle fiber found in muscles of the pig are not yet fully understood.

Post-translational modifications of proteins, including phosphorylation, acetylation, and ubiquitination, likely play crucial roles in the regulation of meat quality (15). With advances in proteomic technologies, many post-translational modifications associated with meat quality have been identified. Recently, phosphorylation proteomics has been used to identify phosphorylated proteins and their modification sites. Weng et al. used a phosphoproteomic approach to identify phosphoproteins associated with meat quality in geese (16). Modifications of protein phosphorylation were identified due to post-slaughter muscle metabolism in pigs and thus could affect meat quality (17). Differential expression of phosphorylated proteins in muscle was found in yak living at different altitudes (18). In addition, studies in mice (19) and humans (20) have found significant differences in proteins between fast and slow-twitch muscle. Deshmukh et al. also showed that proteomic changes occur in fast and slow-twitch muscle during exercise training in humans (20). While many of these reports focused on disease and health-related features of muscle fiber types, the consequences of the modification of phosphorylation in muscle fiber types in porcine muscle is still unknown.

The Duroc breed is usually used as a terminal sire, as it has an excellent growth rate (21), while the Chinese native Meishan pig has a high prolificacy and high-quality pork (22). Therefore, offspring of a cross between Duroc and Meishan pigs were used in the current study. In this study, we examined meat quality, and muscle fiber characteristics from three fast-twitch (biceps femoris, BF) and three slow-twitch (soleus, SOL) pig muscle samples. Phosphoproteins, and their phosphorylation sites, were identified and characterized from these six muscle samples. Differentially expressed phosphorylation sites (DEPSs) between the BF and SOL samples were identified and GO and KEGG enrichment analyses were performed on the proteins containing these sites. Finally, a PPI network analysis was conducted with the phosphoproteins containing these DEPSs. This data provides a basis for mechanisms involving protein phosphorylation that affect pork muscle fiber type transformation and meat quality.

## Materials and methods

### Animals and sample collection

Muscle samples used in these experiments were obtained from three full sib male animals derived from a cross between a Duroc boar and a Meishan sow, with the sibs raised under identical environmental conditions. The animals were slaughtered at an age of 180 days in a standardized commercial abattoir (Jiangsu Sushi Meat Product Co., Ltd., Huaian, China) according to Chinese slaughter guidelines (GB/T 17236-2019), and biceps femoris (BF) and soleus (SOL) muscle tissues collected from each of the three individuals. All collected samples were immediately frozen in liquid nitrogen and stored at −80°C until use. All animal procedures were approved by the Ethical Committee and Experimental Animal Committee of Shenyang Agricultural University.

### Determination of BF and SOL muscle meat quality

The lightness (L*), redness (a*), and yellowness (b*) values of meat color were measured at 45 min after slaughter using a portable Minolta colorimeter (CR-10, Minolta, Japan). The pH value of the meat was determined with a pH meter (Hanna, Thornleigh, NSW, Australia). Drip loss was measured using a bag method following an operational procedure. The shear force was evaluated using a C-LM3 digital tenderness instrument according to the operating instructions.

### Measurement of muscle fiber characteristics

For hematoxylin and eosin (HE) staining, the muscle tissue was fixed in 4% paraformaldehyde, embedded in paraffin, and stained with hematoxylin and eosin. Each tissue section was imaged using an inverted microscope (Olympus, Tokyo, Japan) and the muscle fiber areas were calculated using Image J software (version 1.53).

### Protein extraction

Muscle tissue samples were lysed with a lysis buffer containing 100 mM NH_4_HCO_3_ (pH 8), 8 M Urea, and 0.2% SDS, followed by ultrasonication for 5 min on ice. The lysate was centrifuged at 12,000 *g* for 15 min and the supernatant was collected into a new tube. Each sample supernatant was reduced with 10 mM DTT at 56^°^C for 1 h, and then alkylated with iodoacetamide for 1 h at room temperature. Afterward, each sample was added to pre-cooled acetone and centrifuged at 12,000 *g* for 15 min to collect the precipitate. The pellet was dissolved with a dissolution buffer containing 0.1 M TEAB (pH 8.5) and 6 M urea. Protein concentration was determined using a Bradford Assay kit (Beyotime Biotechnology, Shanghai, China) according to the manufacturer’s instructions. For investigate total protein integrity and purity, 20 μg of protein from each sample was subjected to 12% SDS-PAGE gel electrophoresis. After electrophoresis, the gel was stained with coomassie brilliant blue R-250.

### Trypsin digestion and TMT labeling

Protein samples were digested at 37°C for 4 h by adding trypsin at a trypsin: protein ratio of 1:100 (w/w). Trypsin and CaCl_2_ were then added to each sample at a trypsin: protein ratio of 1:100 (w/w) and incubated overnight. Formic acid was then added to each digest to adjust the pH to less than 3 and centrifuged at 12,000 *g* for 5 min. The supernatants were desalted with C18 columns, washed three times with 0.1% formic acid-3% acetonitrile, and digests eluted with 75% acetonitrile-0.1% formic acid. Peptides were reconstituted with 100 μl of 0.1 M TEAB buffer and added to 82 μl of acetonitrile-soluble TMT labeling reagent (Thermo Fisher Scientific, Waltham, MA, USA) for 2 h at room temperature.

### Phosphopeptide enrichment

A High Select™ Fe-NTA Phosphopeptide Enrichment Kit (Thermo Fisher Scientific, Waltham, MA, USA) was applied to enrich for phosphorylated peptides. Briefly, each lyophilized peptide sample was resuspended in 200 μl binding buffer, and then centrifuged at 12,000 *g* at 4°C for 5 min. The supernatant was then loaded onto an equilibrated spin column, incubated at room temperature for 30 min, centrifuged at 2,000 *g* for 30 s, and then washed three times using washing buffer. Peptides were eluted with elution buffer and centrifuged at 1,000 *g* for 30 s.

### Liquid chromatography tandem-mass spectrometry (LC-MS/MS) analysis

LC-MS/MS analysis was performed with an EASY-nLC™ 1200 UHPLC system (Thermo Fisher Scientific, Waltham, MA, USA) coupled with an Q Exactive HF-X mass spectrometer (Thermo Fisher Scientific, Waltham, MA, USA). Samples were redissolved with buffer A (0.1% formic acid) and injected onto a C18 nano trap column (2 cm × 75 μm, 3 μm). Peptides were separated in an analytical column (15 cm × 150 μm, 1.9 μm), and eluted using a 180 min linear gradient from 6 to 100% of buffer B (0.1% formic acid, 80% acetonitrile) at a flow rate of 600 nl/min. Separated peptides were analyzed using Q Exactive HF-X, with an ion source of Nanospray Flex™ (ESI), spray voltage of 2.3 kV, and ion transport capillary temperature of 320°C. MS scans were acquired in a data-dependent acquisition mode with a scan range of m/z 350–1,500, a resolution of 60,000 (200 m/z), an automatic gain control (AGC) target value of 3 × 10^6^, and a maximum ion injection time of 20 ms. The 40 most abundant ions were fragmented by higher energy collisional dissociation (HCD) and analyzed in MS/MS with a resolution of 45,000 (200 m/z), an AGC target value of 5 × 10^4^, a maximum ion injection time of 54 ms, a normalized collision energy of 32%, an intensity threshold of 1 × 10^4^, and a dynamic exclusion parameter of 20 s.

### Phosphoproteomic data analysis

Raw spectra from each fraction were searched against the *Sus scrofa* uniprot database (188,977 entries) by Proteome Discoverer 2.4 (PD 2.4, Thermo Fisher Scientific, Waltham, MA, USA). Proteome Discoverer 2.4 analysis parameters are as follows: enzyme for digestion: trypsin; missed cleavage sites maximum allowed was 2; precursor mass tolerance of 10 ppm and fragment mass tolerance of 0.02 Da; oxidation methionine, TMT plex of lysine, and phosphorylation of serine (S), threonine (T), and tyrosine (Y) as variable modifications, and carbamidomethyl cysteine as a fixed modification.

Peptide spectrum matches (PSMs) with a confidence level of 99% or higher are considered as plausible PSMs, and proteins containing at least one unique peptide are considered as plausible proteins. Only plausible peptides and proteins with an FDR of less than 1% were retained in this study. Comparative analysis of differences in phosphorylation sites between the two groups was performed using a Student’s *t*-test. Phosphorylation sites with a fold change > 1.2 or < 0.83 and a *p*-value < 0.05 were considered to be differentially expressed phosphorylation sites (DEPSs) based on previous studies (16, 23).

### Motif analysis of phosphorylation sites and analysis of kinase-substrate relationships

To analyze the motifs for the identified phosphorylation site sequences, we used the Motif-X algorithm (version 1.0) (24) to significantly enrich motifs from the phosphorylated peptides. All enrichment analyses were performed based on seven amino acids upstream and downstream of the phosphorylation site with occurrences > 20 and *p*-value < 10^–6^. Further, WebLogo (version 3.5) (25) was used to draw the diagrams generated by the Motif-X analysis. For kinase-substrate relationships, all identified serine, threonine, and tyrosine phosphorylation sites were evaluated with the NetworKIN algorithm (26).

### Gene ontology and KEGG analysis of the phosphorylated proteins

Gene ontology analysis was conducted using the InterProScan 5 program (version 5.22-61.0) (27) against the Pfam database. KEGG annotation was performed using BLASTp program (version 2.2.26) against the KEGG database with an *e*-value ≤ 1e-4. GO and KEGG enrichment analysis were carried out using hypergeometric tests, with the threshold set at *p* < 0.05.

### Construction of protein-protein interaction

We used the STRING database (28) to construct a network of interactions between proteins. In addition, Cytoscape software (version 3.4.0) (29) visualized these protein-protein interactions.

### Subcellular localization of protein

The subcellular localization of the identified phosphorylated proteins were predicted using the Cell-PLoc 2.0 (version 2.0) package (30).

### Statistical analysis

Statistical analyses were conducted using GraphPad Prism 9 (GraphPad Software, La Jolla, USA). Student’s *t*-test was used to calculate the significance between the two groups. All data are expressed as mean ± SEM.

## Results and discussion

### Meat quality and muscle fiber characteristics of BF and SOL muscle

**FIGURE 1:**
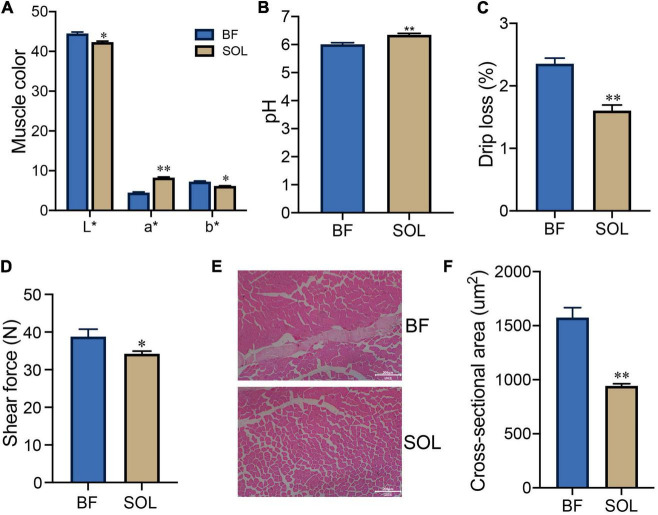
Analysis of meat quality and muscle fiber characteristics of BF and SOL muscle. Determination of BF and SOL meat color **(A)**, pH **(B)**, drip loss **(C)**, and shear force **(D)**. All data were expressed as mean ± SEM (*n* = 3). **(E,F)** HE staining and muscle fiber area analysis of BF and SOL. At least 150 muscle fibers were analyzed in each sample. Scale bars, 200 μm. *P < 0.05 and ^**^P < 0.01.

To demonstrate the difference in meat quality between the fast (BF) and slow (SOL) muscles, we tested these two types of muscle for meat color, pH, drip loss, and shear force traits. Our results showed that the L* and b* values of meat color for SOL were lower than those for BF (*P* < 0.05), while the a* value of meat color for SOL was higher than those for BF (*P* < 0.05; Figure 1A). In addition, the pH of SOL was higher than BF (*P* < 0.05; Figure 1B), while drip loss and shear force of SOL were decreased compared to BF (*P* < 0.05; Figures 1C, D). These results suggest that the meat quality of slow-twitch muscle was significantly improved compared to fast-twitch muscle. We further observed that the muscle fiber area of SOL was smaller than for BF (*P* < 0.05; Figures 1E, F). Our results are consistent with findings in previous studies looking at slow-twitch (psoas major) and fast-twitch (longissimus thoracis) muscles (12). Previous studies have demonstrated that dihydromyricetin and lycopene improve pork quality by regulating the transformation from fast-twitch to slow-twitch (10, 31). Thus, resolving the mechanism for the conversion of muscle fiber types potentially opens a way to improve pork quality.

### Global phosphoproteomic analysis of BF and SOL muscle

**FIGURE 2:**
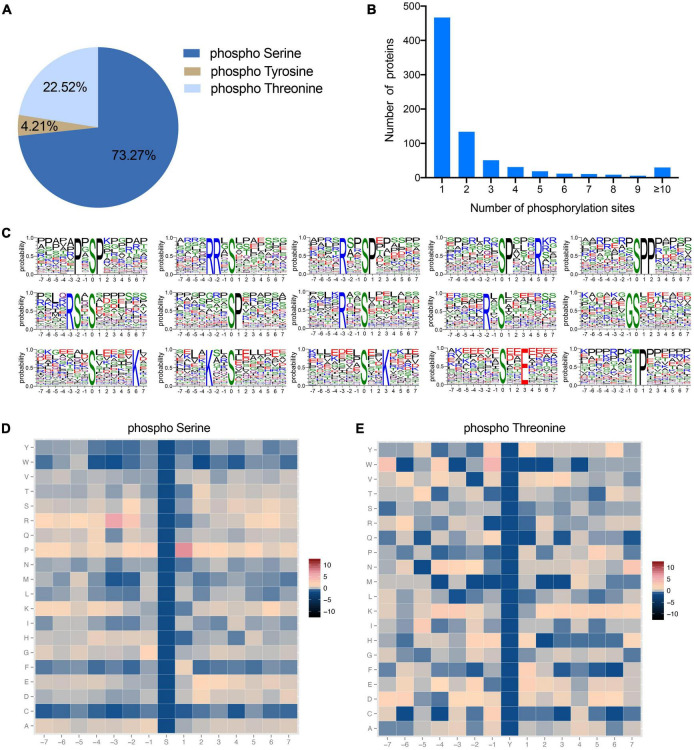
Characterization of identified phosphorylation sites. **(A)** Distribution of amino acids in the identified phosphorylation sites. **(B)** Number of phosphorylation sites in each protein. **(C)** Enriched sequence motifs of phosphorylation sites together with the seven amino acid sites upstream and downstream of the phosphorylation site. The height of each letter represents the probability of finding that amino acids in the motif. The central amino acid represents the phosphorylation site. **(D,E)** Heatmaps of the frequencies of the different amino acids flanking the phosphorylated serine and threonine residues, respectively. Different colors represent the possibilities.

To explore the functional role of protein phosphorylation modifications in fast-twitch (BF) and slow-twitch (SOL) muscle, we first established their phosphoproteome landscape using a TMT-labeled quantitative phosphoproteomic technique. Here, a total of 3,332 phosphorylated peptides were identified, which correspond to 2,327 phosphorylation sites within 770 distinct phosphoproteins (Supplementary Table 1). Several previous studies have identified phosphorylation sites, and their proteins, in pigs based on phosphoproteomic approaches (17, 32). Our study identified a greater number of phosphoproteins, which suggests that the identification of phosphorylation sites in pig proteins is still incomplete. Of the identified phosphorylation sites, the majority occur at serine (S) residues, accounting for 73.27% of the sites, followed by threonine (T) (22.52%) and tyrosine (T) (4.21%) (Figure 2A). This distribution of phosphorylation across these three amino acid residues is consistent with those identified in sheep (33), yak (18), chicken (34), and geese (16). These data suggest that the amino acid residues modified by phosphorylation are relatively similar in different animals. Furthermore, the number of phosphorylation sites on each protein is variable, ranging from 1 to 51 (Figure 2B). A statistical analysis of the phosphoproteins showed that 60.65% of these proteins had only a single phosphorylation site, 17.40% had two phosphorylation sites and the remaining 21.95% had three or more phosphorylation sites (Figure 2B). The motif of phosphorylation site motifs of our phosphoproteins was evaluated using Motif-X software, which showed that 15 conserved motifs could be identified, 14 for serine phosphorylation and 1 for threonine phosphorylation (Figure 2C). The major serine phosphorylation motifs were P×SP, RR×S, R××SP, SP×××R, SPP, RS×S, SP, R××S, R×S, GS, S×××××K, K××S, S××K, and S××E, while the threonine phosphorylation motif was TP, where × is any residue (Figure 2C). The above motifs were also observed in a phosphoproteomic analysis of broiler chicken proteins (34), suggesting that, as expected, phosphorylation modification sites are conserved across animals. A heatmap analysis of the preferences for each of these 15 amino acid sequence motifs revealed that arginine was the preferred amino acid upstream of the phosphorylated serine or threonine residue (Figures 2D, E), while proline was preferred to be located downstream (Figures 2D, E). Protein phosphorylation is catalyzed by protein kinases, which in turn play a role in many biological processes (35). Therefore, we performed kinase-substrate interaction analysis and the results showed that many kinases, including AMPKa2, PDHK1, and CaMKII gamma, could potentially bind to the identified phosphorylation sites, suggesting that these kinases are responsible for regulating protein phosphorylation processes in porcine muscle. Taken together, these data can provide a basis for the identification of potentially phosphorylated proteins in pig muscle tissue.

### Functional analysis of phosphorylated proteins

**FIGURE 3:**
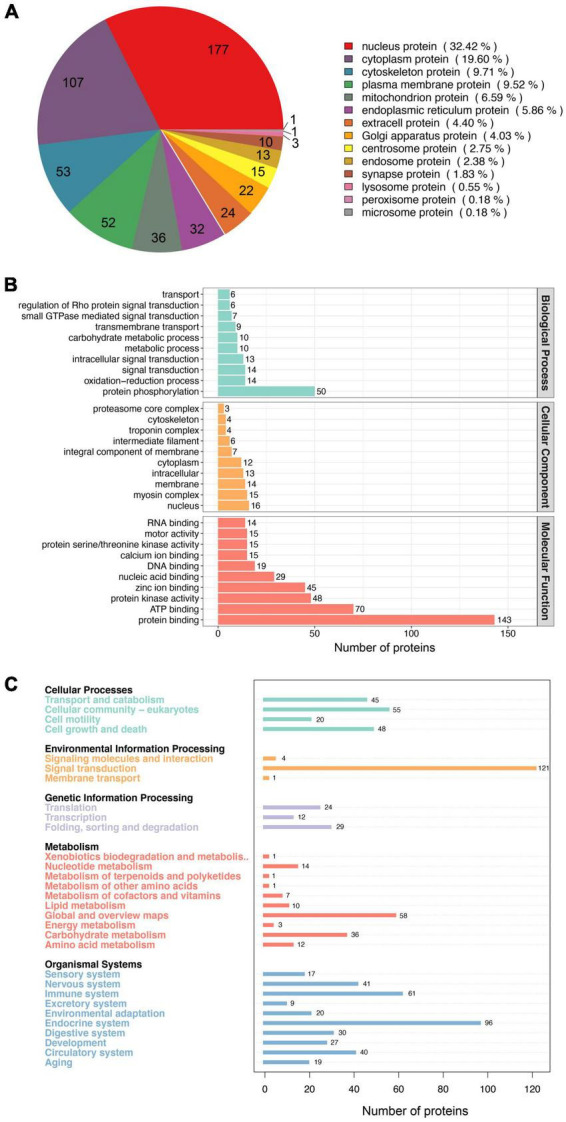
Functional analysis of the phosphorylated proteins. **(A)** Subcellular distribution of the phosphorylated proteins. **(B)** GO annotated classification of the phosphorylated proteins. **(C)** KEEG annotated classification of the phosphorylated proteins.

To understand the functional roles of these 770 phosphorylated proteins in muscle cells, we assessed the predicted subcellular localization of each of these proteins. The phosphoproteins were located in diverse fractions of the cell, with 32.42% predicted to be localized to the nucleus, 19.60% to the cytoplasm, and 6.59% to the mitochondria (Figure 3A). This distribution of phosphoproteins is similar to those seen in a previous study (36). In addition, we annotated the phosphoproteins into 290 GO terms, of which 109, 42, and 139 were in the Biological Process (BP), Cellular Component (CC), and Molecular Function (MF) categories, respectively (Supplementary Table 2). The top 10 BP, CC, and MF terms are shown in Figure 3B. The GO term containing the largest number of proteins in BP is protein phosphorylation, suggesting that the phosphorylated proteins obtained in this study are plausible. The largest number of proteins for GO terms in CC and MF were nucleus and protein binding, respectively. Previous evidence implicates that phosphorylation can create binding sites for certain proteins, thus allowing protein interactions (37). Furthermore, KEGG annotation of these phosphoproteins classified them into 30 level 2 pathways (Figure 3C). Of these, 121 proteins were annotated as Signal transduction pathways. This data provides information that can be used for functional studies of these phosphorylated proteins in pig muscle.

### Identification of differentially expressed phosphorylation sites between BF and SOL muscles

**FIGURE 4:**
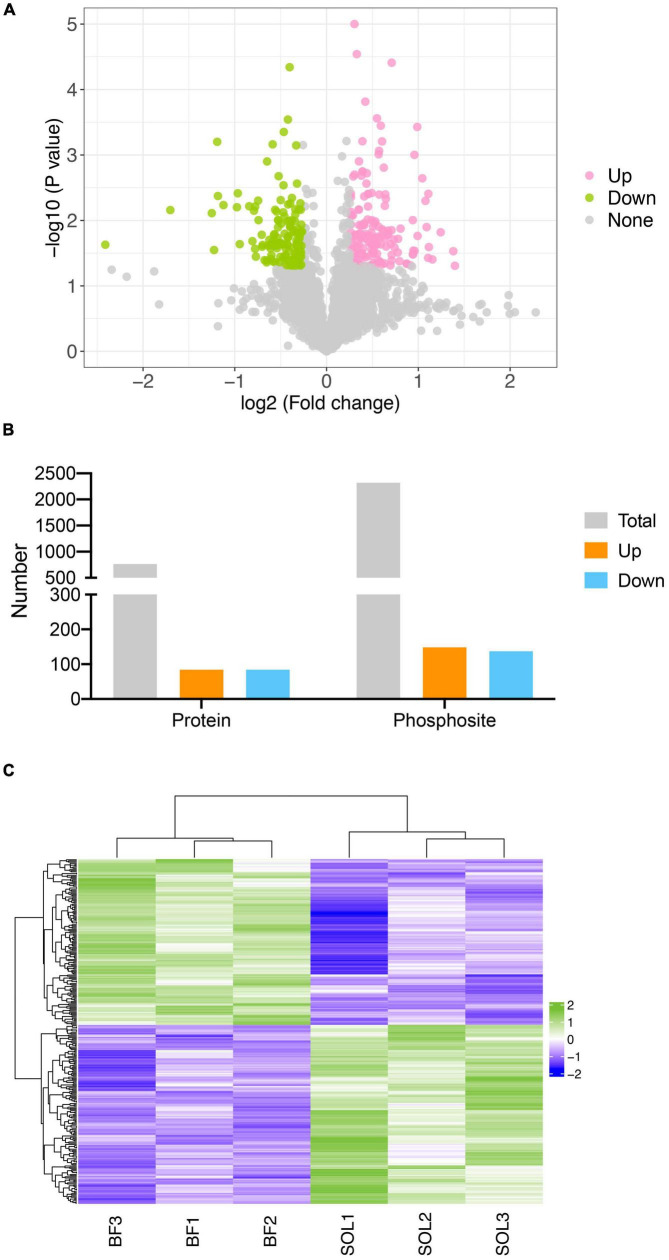
Identification of differentially expressed phosphorylation sites (DEPSs) in BF and SOL muscle tissue. **(A)** Volcano plot showing DEPSs in BF vs. SOL. DEPSs which are significantly up-regulated or down-regulated are indicated in pink and green dots. DESPs without statistical significance are indicated by gray dots. **(B)** Number of total, up- and down-regulated DEPSs and their corresponding phosphorylated proteins. **(C)** Heat map showing the abundance patterns of the DEPSs in BF and SOL.

To examine the involvement of changes in protein phosphorylation in the transformation of muscle fiber types, we identified differentially expressed phosphorylation sites (DEPSs) between BF and SOL muscles. A total of 287 phosphorylation sites were found to be differentially phosphorylated between BF and SOL muscles (Figure 4A), which included 149 up-regulated sites, in 85 proteins, and 138 down-regulated sites, in 85 proteins (Figure 4B). Details on these DEPSs are shown in Supplementary Table 3. The top five up-regulated phosphorylation sites were Ser1348, Thr1492, and Ser742 in Myosin-4 (MYH4), Thr354 in Titin, and Thr481 in Myosin binding protein C1 (MYBPC1) (Supplementary Table 3). The top five down-regulated phosphorylation sites were Ser434 in UTP-glucose-1-phosphate uridylyltransferase (UGP2), Ser152 in Receptor expression-enhancing protein (REEP1), Ser1294 in Titin isoform X6, Ser159 in A0A4X1VV29 (an uncharacterized protein), and Ser67 in Arsenite-resistance protein 2 (Supplementary Table 3). The expression profiles of these DEPSs were quantitated in samples from three BF and three SOL muscles. A heatmap result shows that the identified DEPSs have a very distinct expression pattern in BF and SOL muscles (Figure 4C), suggesting that these DEPSs may be associated with muscle fiber type conversion.

Interestingly, many phosphorylation sites are found on slow- and fast-type sarcomeric proteins. For example, 12 phosphorylation sites are found on the fast-type sarcomeric protein, MYH4, and a single phosphorylation site on slow-type sarcomeric protein MYH7 (Thr446) and fast-type sarcomeric protein tnnt3 (Ser200). These data suggest that post-translational modification of sarcomeric proteins by phosphorylation may be associated with muscle fiber types. The metabolic properties of fast and slow muscle differ due to differences in their glycolytic and mitochondrial oxidative enzymes (38). Here, we found that the key glycolytic enzyme, phosphofructokinase 1 (PFKM) (38), has six DESPs that are significantly down-regulated in SOL muscle. This data suggests that phosphorylation of PFKM proteins may inhibit the conversion of fast to slow muscle by regulating glycolytic process. The calcium-transporting ATPase (ATP2A1), a protein responsible for Ca^2+^ re-uptake in fast muscles (2), has six DESPs that were significantly down-regulated in SOL, which indicated that reduced phosphorylation of ATP2A1 promoted slow muscle development. In addition, nebulin (NEB) protein has six upregulated DEPSs in SOL (Ser393, Ser608, Ser432, Thr6256, and Thr6318). Li et al. demonstrated that slow muscle fibers are significantly increased in the muscle tissue from mice with a conditional knockout NEB (39), suggesting that the six DEPSs in NEB could contribute to the facilitation of a switch from a slow- to fast-twitch muscle. The identified DEPSs are essential for the development of the fast muscle fiber type. The precise molecular mechanisms for this change need to be verified in the future functional studies.

### Functional analysis of proteins with DEPSs

**FIGURE 5:**
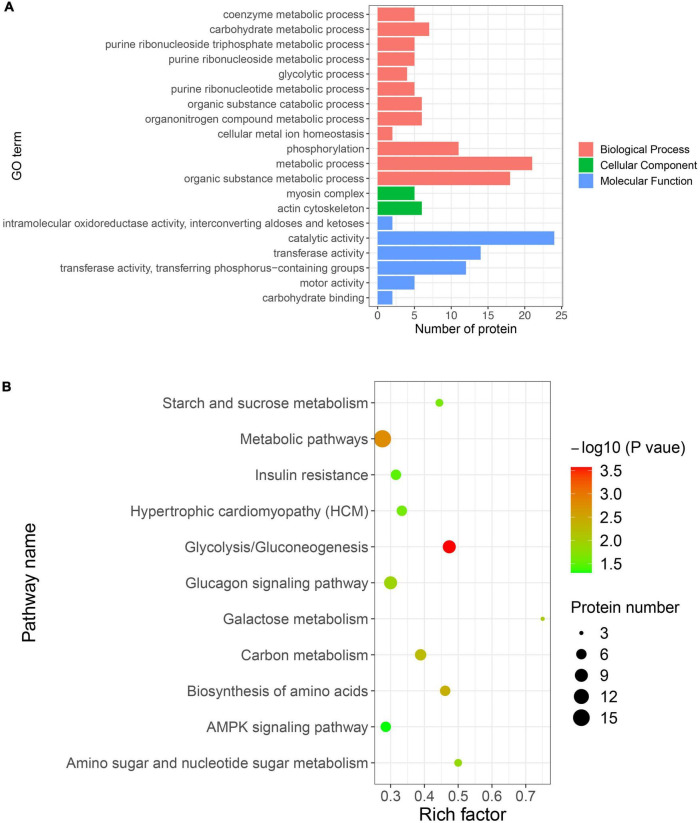
Gene ontology (GO) and KEGG enrichment analyses of proteins with DEPSs. **(A)** Significantly enriched GO terms in biological process, cellular component, and molecular function. The *x*-axis represents the protein number, and *y*-axis represents the GO term. **(B)** Significantly enriched pathways. The *x*-axis represents the rich factor, and *y*-axis represents the pathway. Color and size of dots represent –log10(*p*-value) and the number of proteins, respectively.

To elucidate the functional role of the DEPSs in the transformation of muscle fiber type, proteins with DEPSs were used for GO and KEGG enrichment analyses. The results of GO and KEGG enrichment analyses are shown in Supplementary Tables 4, 5. GO enrichment analysis showed significant enrichment for GO terms associated with the glycolytic process in biological process, myosin complex and actin cytoskeleton in cellular component, and catalytic activity and transferase activity in molecular function (Figure 5A). Increased glycolysis is required for fast-twitch muscle (2), where four glycolytic enzymes (PFKM, PKM, GPI, and PGK1) are enriched. A previous study demonstrated that PGK1, the first ATP-producing enzyme in glycolysis, facilitates the glycolytic process in cells by undergoing modification of its phosphorylation (40). The phosphorylation levels at the DESPs of these glycolytic enzymes were significantly down-regulated in SOL, indicating that glycolytic enzyme activities are inhibited in slow muscle. Also, pathway enrichment analysis of the proteins with DEPSs revealed a number of pathways associated with muscle fiber types, including Glycolysis/Gluconeogenesis, Metabolic pathways, Carbon metabolism, and AMPK signaling pathway (Figure 5B). Some studies have reported that the AMPK pathway can promote fast to slow muscle fiber transformation by activating PGC-1a activity, which in turn stimulates mitochondrial gene expression (41–43). These data suggest that phosphorylated proteins enriched in the AMPK pathway may promote slow fiber development in SOL by activating mitochondrial biogenesis. However, the specific regulatory mechanisms need further evidence. The investigation reported a SNP mutation in the *PRKAG3* gene encoding the AMPK γ3 subunit, which in turn leads to inferior meat quality (44). It might be possible to design AMPK signaling pathway activators, through the phosphorylation sites, that improve pork quality. In conclusion, differentially expressed phosphorylated proteins likely regulate pig fiber type transformation through glycolysis-related processes and the AMPK pathway.

### Protein-protein interaction analysis of proteins with DEPSs

**FIGURE 6:**
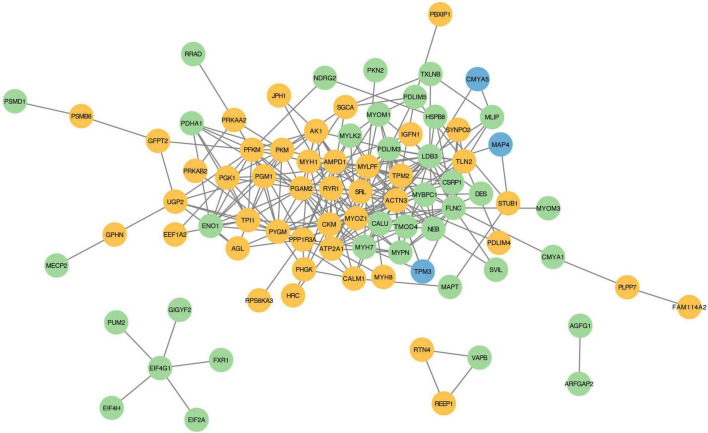
Protein-protein interaction regulatory network of proteins with DEPSs. Each node represents a phosphorylated protein. Green indicates up-regulated, yellow for down-regulated, and blue for both up- and down-regulated proteins. Each edge represents an interaction between proteins.

To examine whether proteins with DEPSs might regulate the different muscle fiber types by binding with each other, we constructed a protein-protein interaction network of the phosphorylated proteins using the STRING database. In our PPI network, there are 84 phosphoproteins that have 268 interactions (Figure 6 and Supplementary Table 6). The network suggests that MYOZ1 is a key protein for regulating myofiber type conversion as it binds to 16 proteins (e.g., TMOD4, PYGM, ATP2A1, MYBPC1, MYH1, and MYLK2). MYOZ1-deficient mice have significantly increased numbers of type I fibers, which causes the conversion of fast-twitch fibers to slow-twitch fibers (45). In the current study, we found that MYOZ1 phosphorylation is down-regulated in slow-twitch SOL muscle. These results suggest that phosphorylated MYOZ1 may promote slow- to fast-twitch muscle phenotype by binding to other proteins. In addition, ACTN3 protein is involved in the positive regulation of fast-twitch muscle contraction and also interacts with many proteins including MYH7, ATP2A1, MYLK2, and TPM2. A study has shown that TPM2 encodes β-Tm (tropomyosin), which regulates muscle contraction by inhibiting actin-myosin protein interactions (46). Seto et al. reported that ACTN3 is an important component of the fast-twitch muscle Z-disk and interacts with other structural muscle proteins (47). Taken together, these data provide fundamental information for the study of phosphorylation-mediated protein-protein interactions that likely regulate porcine muscle fiber transformation.

## Conclusion

In this study, a phosphoproteomic analysis was carried out comparing fast-twitch BF muscle with slow-twitch SOL muscle in pigs. Our phosphoproteomic data identified 287 DEPSs, in 161 proteins, which were mainly enriched in glycolysis-related and AMPK signaling pathways. A PPI analysis resolved a regulatory network of phosphorylated protein-protein regulation for fiber type transformation, particularly glycolytic enzymes. These results provide new insights into the role of protein phosphorylation modification in the transformation of porcine muscle fiber types and meat quality.

## Data availability statement

The mass spectrometry proteomics data have been deposited to the ProteomeXchange Consortium (http://proteomecentral.proteomexchange.org) *via* the iProX partner repository with the dataset identifier PXD036066 (http://proteomecentral.proteomexchange.org/cgi/GetDataset?ID=PXD036066).

## Ethics statement

This animal study was reviewed and approved by the Ethical Committee and Experimental Animal Committee of Shenyang Agricultural University.

## Author contributions

BL and SZ designed the experiments. YH, XT, HL, and ZY performed the experiments. JC, RZ, and WW analyzed the data. YH, XT, and BL wrote the manuscript. DI and BL revised the manuscript. All authors contributed to the article and approved the submitted version.
